# Can Self-Determination Explain Dietary Patterns Among Adults at Risk of or with Type 2 Diabetes? A Cross-Sectional Study in Socio-Economically Disadvantaged Areas in Stockholm

**DOI:** 10.3390/nu12030620

**Published:** 2020-02-27

**Authors:** Nuria Güil Oumrait, Meena Daivadanam, Pilvikki Absetz, David Guwatudde, Aravinda Berggreen-Clausen, Helle Mölsted Alvesson, Jeroen De Man, Kristi Sidney Annerstedt

**Affiliations:** 1 Department of Global Public Health, Karolinska Institutet, 171 65 Solna, Sweden; 2 Department of Food Studies, Nutrition and Dietetics, Uppsala University, 751 22 Uppsala, Sweden; 3 International Maternal and Child Health division, Department of Women’s and Children’s Health, Uppsala University, 752 37 Uppsala, Sweden; 4 Collaborative Care Systems Finland, 00270 Helsinki, Finland; 5 Department of Epidemiology and Biostatistics, School of Public Health, Makerere University, Ggaba Road, Kansanga Box 20000, Uganda; 6 Centre for General Practice, Department of Primary and Interdisciplinary Care, University of Antwerp, 2000 Antwerp, Belgium

**Keywords:** type 2 diabetes, healthy diet, self-determination theory, social support, competence, autonomous motivation, socio-economically disadvantaged, Sweden

## Abstract

Type 2 Diabetes (T2D) is a major health concern in Sweden, where prevalence rates have been increasing in socioeconomically disadvantaged areas. Self-Determination Theory (SDT) is posited as an optimal framework to build interventions targeted to improve and maintain long-term healthy habits preventing and delaying the onset of T2D. However, research on SDT, T2D and diet has been widely overlooked in socio-economically disadvantaged populations. This study aims to identify the main dietary patterns of adults at risk of and with T2D from two socio-economically disadvantaged Stockholm areas and to determine the association between those patterns and selected SDT constructs (relatedness, autonomy motivation and competence). Cross-sectional data of 147 participants was collected via questionnaires. Exploratory Factor Analysis was used to identify participants’ main dietary patterns. Multiple linear regressions were conducted to assess associations between the SDT and diet behaviours, and path analysis was used to explore mediations. Two dietary patterns (healthy and unhealthy) were identified. Competence construct was most strongly associated with healthy diet. Autonomous motivation and competence mediated the effect of relatedness on diet behaviour. In conclusion, social surroundings can promote adults at high risk of or with T2D to sustain healthy diets by supporting their autonomous motivation and competence.

## 1. Introduction

Type 2 diabetes (T2D) is one of the world’s most prevalent and burdening chronic diseases and is within the top ten global causes of death [1,2]. Sweden has experienced a rise in the prevalence of T2D in the last decades and T2D is forecasted to increase from 7% to 10% by 2050 [3], mostly among vulnerable groups living in socio-economically disadvantaged areas [4] characterised by super diversity (i.e., socioeconomic status, country of origin, language, age, sex and educational level) [5].

Maintaining a healthy diet rich in fruits and vegetables prevents and delays the onset of T2D [6,7], and is one of the key components in diabetes prevention and self-management interventions [8,9]. Attempting to change dietary habits often results in short-term adherence, including for people with diabetes or at high risk of developing diabetes [10].

Self-Determination Theory (SDT) represents a broad framework for the study of human motivation that has received considerable attention in behavioural change research. Within the SDT, the Basic Psychological Needs theory posits three universal psychological needs that are involved in individuals’ self-determination: autonomy (being the origin of one’s behaviour), competence (feeling confident and capable with one’s actions) and relatedness (being understood and cared for by others). The satisfaction of these needs constitutes the basis of self-motivation, and consequently, enhances individuals’ (personal) well-being [11]. The influencing role of the social environment is further highlighted in the SDT. In particular, the presence of social support may improve individuals’ basic psychological needs, building the basis of self-determination and enhancing autonomous motivational styles [12]. Full autonomous (self-determined) motivation refers to the engagement in a behaviour that emanates from the self [13]. Individuals who are more autonomously motivated to eat healthily have been shown to find it stimulating and to have more willingness to incorporate new strategies for making healthy meals [11,13].

Previous studies on SDT constructs among patients with T2D have focused primarily on their relationship with glycaemic control (i.e., Hemoglobin A1c levels) among patients with T2D by medication compliance and adherence to a healthy diet and exercise [14,15]. A recent study assessed the association between autonomous motivation, competence and healthcare support on fruits, vegetables and berries intake in Finland [16]. Human behavioural and psychological research has been primarily focused on studies conducted in Western, educated and middle–high-income groups, which ultimately comprise a small part of the global population. The lack of representation of groups with other characteristics along with the implicit assumption of generalisability and universality of certain psychological domains or behavioural phenomena has been criticised in recent years [17].

To the best of our knowledge, there is little research on the relationship between those constructs and dietary patterns within T2D high-risk populations living in socio-economically disadvantaged areas [18]. Despite existing national dietary surveys identifying the dietary patterns among Swedish adults [19], the exploration of dietary patterns within superdiverse socio-economically disadvantaged groups has been widely overlooked, particularly with regard to high-risk/T2D. The findings obtained from our study can provide a window of opportunity to identify strategies for improving lifestyle interventions better aligned with their needs, and eventually ameliorate their physical and mental well-being in the long-term.

This study aimed to: (a) identify the main dietary patterns of a population of adults at risk of or with T2D living in socio-economically disadvantaged areas in Stockholm, and (b) determine the relationship between the Basic Psychological Needs, as outlined in the SDT (relatedness, autonomous motivation and competence) and their dietary patterns. Based on the SDT principles and previous research conducted with T2D, we hypothesized that (a) relatedness, autonomous motivation and competence were associated with health conducive dietary patterns, and (b) autonomous motivation and competence mediate the association between relatedness and participants’ diet behaviour.

## 2. Materials and Methods

### 2.1. Study Design and Procedure

This cross-sectional study used the Swedish baseline data collected between June 2017 and January 2019, as part of the project titled “Self-management approach and reciprocal learning for type 2 diabetes” (SMART2D). The SMART2D project is a multi-country research project focused on the prevention and management of T2D through SDT-based community interventions promoting healthy habits in the framework of diabetes [20]. In Sweden, the study was conducted in two socio-economically disadvantaged districts of Stockholm County suburbs with a high proportion of migrants (≥36%), especially from the Middle East and Africa [21]. These neighbourhoods are characterised by their poor housing conditions, low-income levels and high unemployment rates [22].

#### 2.1.1. Study Participants and Recruitment

Individuals who were aged 30 to 75 years and lived in the community for at least six months before enrolment in the study were eligible to participate. Individuals were excluded if pregnant, diagnosed with heart disease, stroke, peripheral vascular disease or with severe mental disorders affecting cognitive functions.

Community screening was carried out to recruit high-risk participants through local organisations, associations and religious centres. The SMART2D team set up the screening activities in public spaces such as in local libraries, the municipality hall or shopping malls. Each site screened subjects in their study population using the Finnish Diabetes Risk Score (FINDRISC) tool [23]. This tool has been validated in several countries and ethnicities across the world, being the most used risk score in predicting undiagnosed diabetes cases [24,25,26,27,28,29]. Participants with a FINDRISC score above 13 were required to do a point-of-care HbA1c test [30]. If the glucose level was above 38, they were referred to one of the collaborating primary health care (PHC) centres through a written notification.

T2D participants were recruited through facility-based screening in two collaborating PHC centres by a specialised diabetes nurse. Individuals with T2D were also screened by health professionals when they had an appointment at the PHC for unrelated reasons.

#### 2.1.2. Data Collection

All data were collected using the SMART2D questionnaire. This study used questions on demographics and socioeconomic status, diet behaviour, anthropometry measures (height, weight and waist circumstance) and selected SDT components (social support, self-efficacy and self-regulation). The SMART2D questionnaire was administered by a trained interviewer in Swedish, English, Arabic or Somali with the help of an interpreter when needed.

### 2.2. Variables

All measures used in the study are described in Table 1. Social support and self-efficacy scales were used as proxy measures of the SDT constructs relatedness and competence, respectively. The reason lies in their flexibility to adapt to different outcomes and (non-Western) contexts. To assess the SDT components, averaged sum scales for each construct were calculated. Omega coefficient (ω) was used to calculate the scales’ internal consistency and varied from 0.81 to 0.87 (presented in Table 1 by construct), which were regarded as acceptable.

Diet variables were adapted from the World Health Organisation (WHO STEPwise approach to Surveillance (STEPS) Non-Communicable Diseases (NCD) Risk Factor Survey [31]) and were expanded to include other food items that are relevant for multi-ethnic groups and diabetes (e.g., fruits and vegetables, refined and non-refined starches, sugary-sweetened drinks) (Table 1). To report the servings consumed on a typical day, participants were assisted with the visual help of ’show cards’ obtained from the WHO STEPs Survey as well as some show cards explicitly developed for SMART2D. The self-reported serving was multiplied by the number of days reported to have eaten those foods in a typical week and divided by seven to estimate the daily intake of food and drinks. Multivariate imputation by chained equations with predictive mean matching of missing data was used to deal with missing values [32].

### 2.3. Statistical Analysis

Descriptive statistics (medians, inter-quartile range (IQR), means and standard deviation (SD)) were used to obtain participants’ main characteristics. Exploratory Factor Analysis (EFA) with principal factor extraction method was used to identify the main dietary patterns based on the food variables. The adequacy of the data was confirmed based on the value of the Kaiser–Meyer–Olkin (KMO) measure of sampling adequacy (0.56) and Bartlett’s Test (*p* < 0.05). Parallel analysis allowed the selection of the main factors with the highest eigenvalue. Oblique rotation was used to identify factor intercorrelations, and hence, to improve the interpretability of the factors extracted.

Assumptions for ordinary least squares were met. Although some of the variables deviated from the normal distribution, this approach has been recommended [37,38,39]. Variance inflation factors smaller than 10 yielded a low risk of multicollinearity effects of the SDT variables. Bivariate associations between the SDT variables, body mass index (BMI) and dietary patterns were tested using the nonparametric Spearman’s correlation test. Multiple linear regressions were calculated between the dietary patterns and the SDT variables. The socio-demographic variables: age, sex and education, were included to adjust for confounding.

The potential mediating role of autonomous motivation and competence were explored using the four-step approach reported by Baron and Kenny et al. [40]. Path analysis was performed to characterise potential direct and indirect effects between the variables. Since multivariate normality was not held, bootstrapping was used in this last stage of the analysis. Model fit was assessed using the recommended indices, including the comparative fit index (CFI), the root mean square of estimation (RMSEA) and the standardised root mean squared residual (SRMR).

### 2.4. Ethical Approval

The SMART2D study was approved by the Stockholm Ethical Review Board (ID: 2016/2521/31/1).

## 3. Results

Altogether, 147 persons participated in the study, of whom, 59 were diagnosed with T2D and 88 were at high risk of developing the disease. Around 59% and 41% were living in District 1 and 2, respectively. While the participants reported a moderate–high daily intake of fruits and vegetables, the majority were either overweight (49%) or obese (38%). The consumption of non-refined starches was slightly higher than the refined ones. There was a low consumption of tubers and high-starch vegetables compared to non-starchy vegetables. Fish intake was occasional (between once and twice per week). Half of the participants did not consume sugary beverages (Table 2).

### 3.1. Participants’ Dietary Patterns

Parallel analysis suggested two factors describing the dietary patterns of the participants. Items belonging to the first factor fit a construct “healthy dietary pattern”, and to the second one “unhealthy dietary pattern”. The “healthy dietary pattern” construct was characterised by moderate–high consumption of fruits and vegetables, non-refined starches, tubers, starch-vegetables and fish to a lesser extent. The “unhealthy dietary pattern” construct was characterised by a high intake of refined starches and sugary beverages (Table 3). The two factors extracted explained 75.8% and 57.9% of the variability in dietary intake data, respectively. See further indicators in Table A1.

### 3.2. Association between the Self-Determination Theory Constructs and the Dietary Patterns

Competence and relatedness correlated positively with a healthy diet, while autonomous motivation did not (Table A2). Additionally, competence was the only construct that correlated negatively with unhealthy diet and BMI, which was correlated positively with relatedness. Table 4 and Table 5 show that competence and relatedness were positively associated with a healthy diet (R^2^ = 0.06; R^2^ = 0.05, *p* < 0.05), whereas only competence was negatively related to unhealthy diet outcomes (R^2^ = 0.10, *p* < 0.01).

**Figure 1:**
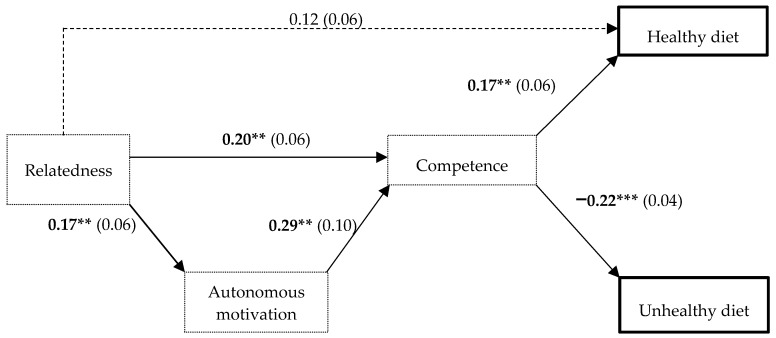
Regression coefficients with standard errors in parenthesis for the direct effects between the SDT and diet pattern variables. Note: Thick squares and dotted squares indicate diet patterns and SDT constructs, respectively. Solid arrows represent significant associations, and dotted arrows represent non-significant associations. Significant β coefficients are bolded. *p < *0*.05; **p < *0*.01; ***p < *0*.001.

Relatedness was associated with autonomous motivation and competence (β = 0.17; β = 0.20, *p* < 0.01), which were associated with each other (β = 0.29, *p* < 0.01). As shown in Figure 1, autonomous motivation and competence acted as mediators at different levels. First, with an indirect effect, autonomous motivation partially mediated the effect of relatedness on competence. Second, competence mediated the association between relatedness and a healthy diet and mediated the relation between autonomous motivation and a healthy diet. With regards to unhealthy dietary patterns, competence also played a mediating role between this outcome and the other SDT constructs (relatedness and autonomous motivation). Table 6 shows the indirect effects of the path analysis model. CFI values exceeding 0.95, SRMR ≤ 0.06 and RMSEA ≤ 0.09 indicated an acceptable model fit.

## 4. Discussion

This study is the first attempt of exploring dietary patterns and the SDT factors related to them of a population with high risk of and with T2D living in socially disadvantaged areas in Stockholm. The results showed that participants who reported a healthy diet were the ones who felt more competent and supported by their social surroundings.

Most of the study participants were overweight or obese, as previously documented in multi-ethnic groups [41,42]. The healthy dietary pattern obtained in this study is similar to the one identified in a recent Swedish National Dietary survey, with positive loadings on fruit, vegetables, fish and non-refined cereals [19]. The unhealthy pattern in our study resembles the ‘Western-type’ dietary pattern identified in previous studies in other high-income countries, and characterised by food products high in energy, such as sugar-sweetened beverages and refined bread and cereals [43]. Interestingly, its homologous in Sweden (categorised as “Swedish traditional” pattern in the Swedish National Dietary survey) slightly differs from our unhealthy pattern since it did not load on sugary drinks [19].

Competence was the SDT construct most strongly associated with both diet patterns. This was interpreted as the participants who felt more confident in maintaining a healthy diet were more likely to follow a healthy dietary pattern. This result is consistent with other studies that highlighted the importance of self-efficacy as a predictor of adults’ fruits and vegetable intake [44,45]. On the other hand, participants who felt less confident in having a healthy diet were more likely to have an unhealthy dietary lifestyle, which is also consistent with previous findings [45,46,47]. Perceived competence is highly important for healthy dietary pattern and our results suggest that it could be boosted through supporting autonomy and relatedness. Previous studies have demonstrated that social support is instrumental in promoting activities that decrease the risk of T2D such as fruits and vegetable intake [16], smoking cessation [48] and physical activity [49]. According to SDT, psychological needs support consists of providing (a) unconditional support and care (relatedness support), (b) alternatives with meaningful reasons for change while respecting someone’s values and personal choices (autonomy support) and (c) overcoming barriers and helping in fixing plans and learning new skills (competence support). When these mechanisms are in place, people will be motivated to eat healthier in a more autonomous way which will then lead to more sustainable behaviour change [11,50].

Unexpectedly, we did not detect any direct association between autonomous motivation and diet patterns. This result is inconsistent with previous studies that obtained strong correlations between autonomous motivation and fruits and vegetable intake [16,51]. A reason for this inconsistency could be the cultural diversity found in our study population and the different aspects of food socialisation they represent (cooking and eating behaviours including different commensality practices) [52], which could influence the sources of the regulation when following a healthy diet [53,54].

Although few values of the path analysis did not reach significance, we did find some suggestion in this population that autonomous motivation may have a mediating role between perceived relatedness and competence, which also mediated the association between autonomous motivation and the dietary patterns, as it is suggested in other studies on SDT [55]. The SDT posits that autonomous motivation fosters competence because when people feel intrinsically engaged, they are willing to search and learn new skills in order to maintain a healthy diet [11,13,55]. Thus, social surroundings (relatives and healthcare professionals) can promote a healthy diet among adults at high risk of and with T2D by supporting their autonomous motivation and competence.

### Strengths and Limitations

Our interview-administered questionnaires allowed us to utilise visual tools such as the ‘showcards’ that assisted participants to report data closer to their reality. The questions were clear, precise and included examples, which minimised misunderstandings. The availability of the questionnaire in different languages, along with the collaboration of interpreters, contributed to obtaining more reliable information. All these helped to decrease the risk of social desirability bias and recall bias entailed in the data collection method [56]. However, social desirability bias should still be considered in the interpretation of the healthy dietary indicators obtained in this study. Exclusion of people suffering from other non-communicable diseases, as mentioned in the methods section, may have diluted the influence of multimorbidity in our study population.

We used proxy measures for the competence and relatedness constructs in order to increase the measurement validity of the SDT components in our study population. However, the small sample size may have hampered the identification of a significant relationship between autonomous motivation and the dietary patterns. Furthermore, the nature of the study design (cross-sectional) only allows us to describe the associations of exposures and outcomes at the same point in time; thus, the directionality of the relations can only be suggestive. However, it is reasonable to believe that perceived social support may influence participants’ eating behaviour through perceived autonomous motivation and competence (and not the other way around).

To our knowledge, this is the first study to detect dietary patterns extracted from an EFA as outcome measurement compared to previous studies, which were limited to the use of fruits and vegetable intake [16] or diet self-care scales [57]. In nutrition epidemiology research, the use of variables that result from dietary patterns has been raised to show a more holistic approach of the nutritional aspects of one individual instead of assessing the intake of one or two single food items [43]. Our dietary patterns explained a substantial amount of the total variance in our study population’s dietary intake (54%–75%), which is a strength of this study. However, this is still an incomplete version of participants’ eating habits since no questions regarding other food items relevant to T2D, such as meat, fats, salt intake or ways of cooking, were included [58,59,60,61]. Future intervention trials addressing healthy dietary adherence by targeting the motivational constructs analysed in this study are needed. The use of dietary patterns that incorporate further information about eating and cooking habits and other elements such as weight management should also be considered.

## 5. Conclusions

Globally, those at the highest risk of developing T2D are populations in low- and middle-income countries, migrants, minorities and socio-economically disadvantaged groups in high-income countries. This study is a first attempt of exploring dietary patterns and their relation to the SDT in a superdiverse socio-economically disadvantaged population at high risk of and with T2D living in Sweden. The findings of this study could be applicable to other socio-economically disadvantaged areas in high-income countries with similar health system provisions.

Competence was the strongest construct associated with a healthy diet, which suggests that participants who felt more self-efficacious were the ones who consumed healthy foods. The effect of relatedness on dietary habits was mediated by competence, in line with the SDT process model. Relatives, friends and healthcare professionals can promote a healthy diet of adults at high risk of or with T2D by focusing on supporting their competence.

It is worth stressing the super diversity and the socioeconomic status of our study population. The results highlight the importance of enhancing the sense of community belonging, integration and trust within those groups. Therefore, close collaborations should be strengthened between different stakeholders, including research and healthcare professionals, policymakers and local associations. More efforts should be targeted in exploring interventions that support high-risk and T2D patients’ needs (relatedness, autonomy and competence) to improve their dietary habits and to prevent or delay the onset of the disease.

## Figures and Tables

**Table 1:** Variables used in the study.

**SDT Variables**	
Relatedness (i.e., social support)	The social support scale developed by Sallis et al. [33] was used as a proxy measure of perceived relatedness. It was adapted and validated in several different contexts and consisted of a five-item scale measuring the frequency of participation and involvement received from friends, family and relatives to maintain a healthy diet (range: 1 = never, 2 = less than once a week, 3 = once a week 4 = more than once a week; ω = 0.87). Example item: How often have people close to you (friends, family or relatives) eaten healthy food with you?
Autonomous Motivation	Four items were selected from the Autonomous Regulation Scale of the Treatment Self-Regulation Questionnaire (TSRQ), guided by factor loadings from a validation study by Levesque et al. [34] (range: 1 = strongly disagree, 5 = strongly agree; ω = 0.81). Example item: Would you maintain a healthy diet because you personally believe is the best thing for your health?
Competence (i.e., self-efficacy)	Five-item scale adapted from the Perceived Self-Efficacy Scale [35] was used as a proxy measure of perceived competence measuring their capacity of maintaining a healthy lifestyle under specific conditions (barriers) (range: 1 = strongly disagree, 5 = strongly agree; ω = 0.82) [36]. Example item: Do you think you can maintain a healthy diet even if you need to change how you cook at home?
**Diet Variables**	
Fruits	In a typical week, on how many days do you eat fruit? How many servings of whole/cut or small (fresh or frozen)/canned fruit/dried fruits do you eat on a typical day?
Vegetables	In a typical week, on how many days do you eat vegetables like tomatoes, carrots, onions, etc. (excluding tubers and high-starch vegetables such as cassava, potatoes, matoke, yams, sweet potatoes)? How many servings of cut (fresh or frozen)/canned/uncooked leafy vegetables do you eat on a typical day?
Refined starches ^1^	In a typical week, how many days do you eat refined starch products, (such as white rice, pasta, white bread, maize meal, cassava flour meal, pap)? How many servings of white bread and other refined starch products do you eat on a typical day?
Non-refined starches ^1^	In a typical week, how many days do you eat non-refined starch (such as brown rice, whole grain pasta, wholegrain cereal, samp or wholemeal)? How many servings of non-refined bread and other non-refined starch products do you eat on a typical day?
Tubers and high-starch vegetables	In a typical week, how many days do you eat tubers and high-starch vegetables (such as cassava, potatoes, matoke/plantain, yams, sweet potatoes)? How many servings of tubers and high-starch vegetables do you eat on a typical day?
Fish	In a typical week, how many days do you eat fish? How many servings of fish do you eat on one of those days?
Sugary drinks ^2^	In a typical week, on how many days do you drink sugar-sweetened beverages (such as sodas, and other non-carbonated commercially prepared fruit drinks)? How many servings of sugar-sweetened beverages do you drink on a typical day?

^1^ Reported in grams (one serving of bread = 28 gr, one serving of starches = 80 gr). ^2^ Reported in mL. SDT: Self-Determination Theory.

**Table 2:** Summary of socio-demographic factors and daily diet habits of the study participants (*n* = 147).

Socio-Demographic Factors	*n* (%)	
Sex		
Male	59 (40)	
Female	88 (60)	
Education		
Mandatory education (0–10 years)	19 (13)	
11 years or vocational training	83 (56)	
University studies	45 (31)	
Employment		
Employed	79 (54)	
Unemployed/Unpaid/Supported by social services	37 (25)	
Retired	31 (21)	
Marital status		
Single	71 (48)	
Married/Co-living	76 (52)	
Country of birth		
Europe	59 (40)	
Outside Europe	88 (60)	
**Other sociodemographic variables**	**Median**	**IQR^1^**
Age (years)	57	47–64
Household income (SEK^2^/month)	30,000	14,500–40,000
**Food variables** (**average daily consumption**)		
Fruits (in servings)	2	0.86–3
Vegetables (in servings)	2	1–3
Refined starches (in grams)	46	16–136
Non-refined starches (in grams)	58	15–136
Tubers and high starch vegetables (in servings)	0.29	0. 14–0.57
Fish (in servings)	0.29	0. 14–0.43
Sugary drinks (in mL)	0	0–107

^1^ IQR: Interquartile Range. ^2^ SEK: Swedish Krona (Currency of Sweden).

**Table 3:** Food grouping factor loadings by dietary pattern.

Food Items	Healthy ^1^	Unhealthy ^2^	Uniqueness
Fruits	0.41	−0.05	0.80
Vegetables	0.59	0.00	0.64
Non-refined starch products	0.25	−0.09	0.78
Refined starch products	−0.12	0.43	0.82
Tubers and high-starch vegetables	0.29	0.39	0.80
Fish	0.21	−0.02	0.93
Sugary drinks	−0.05	0.45	0.78

Healthy dietary pattern^1^ versus Unhealthy dietary pattern ^2^.

**Table 4:** Linear regression models on the association of healthy dietary pattern and SDT variables.

	Crude Model		Adjusted Model	
SDT Construct	β	95 % CI ^1^	β	95 % CI
Competence	0.21 **	(0.08, 0.33)	0.19 **	(0.06, 0.32)
Relatedness	0.15 **	(0.04, 0.27)	0.16 **	(0.04, 0.27)
Autonomous motivation	0.04	(−0.12, 0.19)	0.03	(−0.13, 0.18)

^1^ CI: Confidence Interval. Adjusted model controlling for the following confounders: age, sex and education *p < 0.05; **p < 0.01; ***p < 0.001.

**Table 5:** Linear regression models on the association of unhealthy dietary pattern and SDT variables.

	Crude Model		Adjusted Model	
SDT Construct	β	95 % CI ^1^	β	95 % CI
Competence	−0.21 ***	(−0.34, −0.11)	−0.21 **	(−0.32, −0.09)
Relatedness	−0.07	(−0.17, 0.04)	−0.07	(−0.18, 0.03)
Autonomous motivation	−0.04	(−0.18, 0.10)	−0.04	(−0.18, 0.11)

^1^ CI: Confidence Interval. Adjusted model controlling for the following confounders: age, sex and education *p < 0.05; **p < 0.01; ***p < 0.001.

**Table 6:** Indirect effects of the path analysis model.

Dependent Variable	Independent Variable	β	Bootstrap Standard Error	*p* Value
Indirect effects via Autonomous Motivation				
Competence	Relatedness	0.05	0.03	0.066
Indirect effects via Competence				
Healthy diet	Autonomous Motivation	0.05	0.03	0.055
Healthy diet	Relatedness	**0.04**	0.02	**0.03**
Unhealthy diet	Autonomous Motivation	**−0.07**	0.03	**0.01**
Unhealthy diet	Social support	**−0.06**	.02	**0.00**

Significant β coefficients are bolded.

## Appendix A

**Table A1:** Principal factors extracted in the Exploratory Factor Analysis.

Factor	Eigenvalue	Variance	Proportion
Eating healthy	0.81	0.76	0.94
Eating unhealthily	0.50	0.58	0.72

**Table A2:** Spearman correlation coefficients between SDT variables, dietary patterns and body mass index (BMI).

Variables	Median	IQR	Possible Range	1	2	3	4	5	6
1. Competence	4.17	3.5–4.67	[1;5]	-					
2. Relatedness	2.2	1.63–3.2	[1;4]	0.30 ***	-				
3. Autonomous motivation	4.75	4.25–5	[1;5]	0.31 ***	0.23 **	-			
4. Healthy diet				0.28 ***	0.23 **	0.06	-		
5. Unhealthy diet				−0.36 ***	−0.12	−0.04	−0.39 ***	-	
6. BMI	29	26.22–32.21		−0.17 *	0.17 *	0.00	−0.06	0.14	-

BMI scores are in kilograms per square meter. IQR: Inter-quartile range **p* < 0.05; ***p* < 0.01; ****p* < 0.001
